# Supplementary material for: Inositol pyrophosphate catabolism by three families of phosphatases regulates plant growth and development
Source: PLoS Genet. 2024 Nov 12;20(11):e1011468. doi: 10.1371/journal.pgen.1011468 (PMC11611267; doi:10.1371/journal.pgen.1011468)
Supplement: Attachment — Submitted filename: Laurent_response_to_reviewers.pdf [file pgen.1011468.s013.pdf]

Dear Dr Hothorn,

Thank you very much for submitting your Research Article entitled 'Inositol pyrophosphate catabolism by three families of phosphatases controls plant growth and development' to PLOS Genetics.

The manuscript was fully evaluated at the editorial level and by independent peer reviewers. The reviewers appreciated the attention to an important problem, but raised some concerns about the current manuscript. Based on the reviews, we will not be able to accept this version of the manuscript, but we would be willing to review a revised version. We cannot, of course, promise publication at that time.

Should you decide to revise the manuscript for further consideration here, your revisions should address the specific points made by each reviewer, especially the first major comment of reviewer #1 and #3 and a more detailed analysis of cell-wall changes (reviewer #3). In our view testing enzymes emerging from the RNAseq analysis is not necessary. We will also require a detailed list of your responses to the review comments and a description of the changes you have made in the manuscript.

author response

Dear editor, dear Caroline, dear Claudia,

we have revised our manuscript according to your and to the reviewers suggestions (changes in the text are highlighted in red). In brief,

(1) we have performed additional *in vitro* enzyme assays that further substantiate that our enzymes are specific inositol pyrophosphate phosphatases (**reviewer #1 major point #1**, **reviewer #3 major point #1**).

(2) we have quantified other inositol phosphate and polyphosphate levels in response to **reviewer #1 major point #3**.

(3) we have performed additional histological analyses for our different Marchantia mutants in response to **reviewer #3 major point #3**.

(4) we have experimentally tested if nitrate starvation affects cell wall architecture in Marchantia (it does) and if these changes are signaled via PP-InsP pool changes (not consistently) in response to **reviewer #3 major point #4**.

(5) We have revised the text to make our findings more accessible in response to **reviewer #2**.

(6) We have corrected several references and a statement regarding auxin signaling in response to comments on our preprint. The revised discussion statement reads (line 418-421): "AtTIR1 has recently been shown to interact with AtITPK1, and thus may bind the AtITPK1 reaction product 5-InsP$_7$ *in planta* (Laha *et al*, 2022). Notably, 5-InsP$_7$ levels are increased in our Mp*pfa-dsp1*^{ge} and Mp*vip1*^{ge} plants, which in turn may alter TIR1-mediated auxin responses (Figure 3G)."

We hope that you will find our revised manuscript to be suitable for further consideration at Plos Genetics.

On behalf of the authors, Michael

###

Please be aware that our data availability policy requires that all numerical data underlying graphs or summary statistics are included with the submission, and you will need to provide this upon resubmission if not already present. In addition, we do not permit the inclusion of phrases such as "data not shown" or "unpublished results" in manuscripts. All points should be backed up by data provided with the submission.

author response

We now include all raw data as .xls files and have added Supplementary table 1 reporting all primer sequences in the revised submission.

###

Reviewer's Responses to Questions

Comments to the Authors:

Reviewer #1: This manuscript describes a substantial body of work, employing a variety of approaches that are executed with great skill. The premise of the manuscript is that a group of molecules, inositol pyrophosphates, regulate plant physiology - some aspects of which have not been described before. This premise makes the assumption that the only inositol phosphates altered by molecular genetic manipulation of two families of phosphatase are, effectively, inositol pyrophosphates. The manuscript characterizes members of two families of phosphatases, PFA-DSP and NUDT, and knock-outs thereof in Marchantia (predominantly) to draw its conclusions. The characterization of the gene products (enzymes) is tested with inositol pyrophosphate substrates only. These experiments are performed with substrate at levels 100-1000 fold physiological as demanded by a low-sensitivity technique such as NMR. One alternative is the use of radioactive substrates. The use of NMR is not inappropriate, the data are robust and the interpretations are straightforward. Numerous studies have shown that inositol pyrophosphates are a vanishingly small proportion of the inositol phosphate content of plant tissues. How much so, is illustrated by the ratioing of inositol pyrophosphate content to InsP6 in (for example) Figure 3. They are less than 0.01-1%.

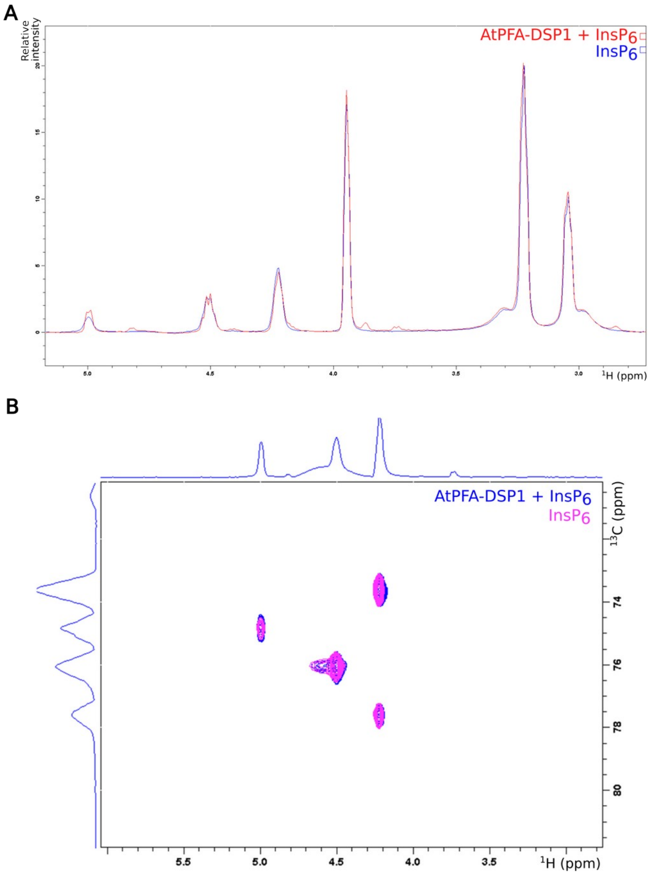

Reviewer Figure 1

(A) Pseudo-2D-BIRD-^{1}H-^{13}C-HMQC spectra as described in Harmel et al., 2019 of the products of 1 uM AtPFA-DSP1 incubated with InsP6 9red trace) or InsP6 alone (blue trace) after 7 h of incubation at 37°C. (B) Overlay of two 2D-^{1}H-^{13}C-HMQC spectra of the products of 1 uM of AtPFA-DSP1 with InsP6 (blue trace) or InsP6 alone (magenta trace) after 7 h of incubation at 37°C.

Reviewer #1 major point #1:

This reviewer thinks it likely that among the many inositol phosphates identified in plants, but not measured in this manuscript, that there might be other candidate substrates. This reviewer suggests that the authors test their enzymes against InsP6 (1mM would be less than 100-fold physiological).

author response

We used [$^{13}C_6$]-labeled substrates in NMR pseudo-2D spin-echo difference phosphatase assays, which allow us to directly monitor PFA-DSP1 or NUDT-meditated conversion of PP-InsP substrates into specific PP-InsP or inositol (poly)phosphate reaction products. Thus, also hydrolysis of $InsP_6$ would be readily detected, but was not observed in the presence of either AtPFA-DSP1, AtNUDT17, MpPFA-DSP1 or MpNUDT1 (Figure 1A,B, Supplementary Fig 2; Figure 3A,B, Supplementary Figure 6). Based on the reviewer's suggestion, we have repeated the assay with a very high enzyme concentration of 1µM AtPFA-DSP1 and in the presence of 1 mM $InsP_6$ substrate in a time course experiment at 37 ºC. No $InsP_6$ hydrolysis was observed even with a high excess of substrate (reviewer Figure 1A,B). These findings are consistent with earlier reports that have characterized fungal and plant PFA-DSP enzymes as specific inositol 5-pyrophosphate phosphatases (Steidle *et al*, 2016, 2020; Wang *et al*, 2018, 2022; Gaugler *et al*, 2022; Sanchez *et al*, 2023). As outlined in the introduction, members of the diadenosine and diphosphoinositol polyphosphate phosphohydrolase subfamily of NUDIX hydrolases have somewhat broader substrate specificities. Yet all known enzymes act on pyrophosphate-moiety containing substrates (Ingram *et al*, 1999; Safrany *et al*, 1999; Garza *et al*, 2009; Lonetti *et al*, 2011; Kilari *et al*, 2013; Márquez-Moñino *et al*, 2021; Zong *et al*, 2021) in line with our NMR enzyme assays, which do not show any AtNUDT17, AtNUDT13 or MpNUDT1-catalyzed conversion of $InsP_6$ to lower phosphorylated inositol phosphates.

###

Reviewer #1 major point #2:

Where measurements of effect of molecular genetic intervention on inositol pyrophosphates are shown (Figure 1, 3) it would be less confusing to the reader to show data for InsP6 content (in uM per g fresh wt) beside the data for inositol pyrophosphates (in the same units), rather than ratioing to InsP6 values that are shown (with different units) in Supplementary figures.

author response

Thank you for this suggestion. We have replotted all inositol phosphate and poly/pyrophosphate levels in µM per g FW as requested, and have included the levels for $InsP_6$ in revised Figures 1F and 3F. The corresponding statements in the result section now read (lines 189-194, changes in red): "We found $InsP_6$ levels in *AtPFA-DSP2* OX and *AtNUDT17* OX to be similar to wild type, while *nudt17/18/21* plants had two times as much $InsP_6$ (Figure 1F). *AtPFA-DSP2* OX lines showed reduced levels of 5-$InsP_7$ and 1,5-$InsP_8$, in agreement with the inositol 5-pyrophosphate phosphatase activity of this enzyme *in vitro* (Figure 1A, B, F). Consistent with our biochemical assays, *AtNUDT17* OX lines also showed reduced 5-$InsP_7$ and 1,5-$InsP_8$ levels (Figure 1A, B, F). Higher levels of 1-$InsP_7$ were observed in *nudt17/18/21* plants (Figure 1F)."

and line 271: "All mutants contained somewhat reduced levels of $InsP_6$ (Figure 3G)."

###

Reviewer #1 major point #3:

Is there a reason why measurements of inositol phosphates/pyrophosphates are restricted to the limited set of species shown in Figures 1 and 3)? Have the authors looked at other species?

author response

Our initial analysis focused on the known inositol pyrophosphate species in plants, which represent the substrates and reaction products of the inositol pyrophosphate phosphatases under investigation (compare point #1 above). As per the reviewer's suggestion, we now report the cellular levels of additional inositol phosphates, namely inositol trisphosphate ($InsP_3$), inositol tetrakisphosphate ($InsP_4$) and inositol pentakisphosphate ($InsP_5$) in our revised Supplementary Figures 5A and 8A. We have added the following statements to the results section (lines 195-197): "With the exception of inositol trisphosphate ($InsP_3$), which was higher in *AtPFA-DSP2* OX and *AtNUDT17* OX compared to wild type, the pools of other inositol phosphates were largely unchanged in our different genotypes (Supplementary Figure 5)."

and lines (272-273):"$InsP_3$ levels were reduced in Mp*pfa-dsp1*^{ge}, while the pools of other inositol phosphates were similar to Tak-1 (Supplementary Figure 8A)."

The method section and figure panels / legends for Supplementary Figs 5A, 5B and 8A have been updated accordingly.

In line with our observations, increased $InsP_3$ levels have been previously reported in AtPFA-DSP1 ox lines, by SAX-HPLC analysis of [^{3}H]-*myo*-inositol labeled seedlings (compare Figure 6A in (Gaugler *et al*, 2022). We have revised the statement in our discussion accordingly (lines 398-400): "Consistent with our study, overexpression of *AtPFA-DSP1* in tobacco and in Arabidopsis resulted in reduced $InsP_7$ and increased $InsP_3$ pools (Gaugler *et al*, 2022). (Figure 1F, Supplementary Figure 5A)."

Notably, the growth phenotypes observed in both Arabidopsis and in Marchantia do not correlate with changes in $InsP_3$ pools: $InsP_3$ levels are 3-fold elevated in *AtNUDT17* OX plants (Supplementary Figure 5A), which do not exhibit the stunted growth phenotypes observed in *AtPFA-DSP2* OX or *vih1 vih2 phr1 phl1* plants, which have either slightly elevated or wild type-like $InsP_3$ pools, respectively (Figure 1C). Likewise, in *M. polymorpha* the vertical thallus growth phenotype is observed in both Mp*pfa-dsp1*^{ge} and Mp*vip1*^{ge} mutants (Figure 3A), but $InsP_3$ levels are reduced only in Mp*pfa-dsp1*^{ge}, while Mp*vip1*^{ge} plants are similar to Tak-1 (Supplementary Figure 8A). Based on these experiments, our conclusion remains that the observed gain and loss-of-function growth phenotypes in Arabidopsis and Marchantia correlate with changes in PP-InsP levels (and with the biochemical activities of the enzymes used in this study, see above), and not with changes in $InsP_3$.

Why certain genotypes contain altered levels of $InsP_3$ (and $InsP_6$, see next point below) remains to be investigated: *AtPFA-DSP2* OX and At*NUDT17* OX have reduced 5-$InsP_7$ and 1,5 $InsP_8$ pools, and higher $InsP_3$ levels. Mp*pfa-dsp1*^{ge} plants had higher levels of 5-InsP7, wild type-like levels of 1,5-$InsP_8$ and reduced $InsP_3$ pools. It thus seems possible that reduction in 5-$InsP_7$ may result in larger $InsP_3$ pools and *vice versa*. We find several PLCs to be differentially expressed in our *AtPFA-DSP2* OX,

At*NUDT17* OX and Mp*pfa-dsp1*^{ge} RNAseq experiments, but we feel that it goes beyond the scope of this study to attempt to dissect the molecular connections between inositol pyrophosphate and inositol phosphate metabolism and signaling.

###

Reviewer #1 major point #4:

The changes in $InsP_6$ measured are much greater (100's of uM) than those for inositol pyrophosphates (fractions of uM), this needs discussing.

author response

Indeed, using the CE-ESI-MS method to quantify TiO_2 bead-extracted InsPs/PP-InsPs, we find $InsP_6$ pools to be 2fold increased in tissue from *nudt17/18/21* plants in Arabidopsis (revised Figure 1F). In Marchantia, we find that $InsP_6$ levels are moderately reduced in all genotypes when compared to the Tak-1 wild type. We have added the following statements to the results section (lines 189-191): "We found $InsP_6$ levels in *AtPFA-DSP2* OX and *AtNUDT17* OX to be similar to wild type, while *nudt17/18/21* plants had two times as much $InsP_6$ (Figure 1F).

and (line 271): " All mutants contained somewhat reduced levels of $InsP_6$ (Figure 3G)."

Why the overexpression or deletion of different inositol kinases and pyrophosphatases alters the cellular $InsP_6$ pools in both Arabidopsis and Marchantia we currently find difficult to rationalize: In Marchantia, the inositol polyphosphate biosynthesis and storage pathways have not been characterized at the genetic or biochemical level. Neither the SAX-HPLC nor the CE-ESI-MS method allows to distinguish between the cytoplasmic and vacuolar pools of $InsP_6$. In Arabidopsis, alterations in PP-InsP levels may affect $InsP_6$ biosynthesis, $InsP_6$ transport/vacuolar storage, or $InsP_6$ remobilization / degradation. It was previously shown that *atipk1-1 atipk2*β*-1* double mutants contain very low levels of $InsP_6$ (Stevenson-Paulik *et al*, 2005). However, our RNAseq analysis of *nudt17/18/21* plants, which have twice as much $InsP_6$ compare to wild-type (Figure 1F), shows no expression differences for either AtIPK1, AtIPK2β or any other inositol phosphate kinase. This suggests that changes in PP-InsP levels do not transcriptionally regulate key $InsP_6$ biosynthetic genes. Regarding $InsP_6$ transport/storage, it has been reported previously that deletion of the putative $InsP_6$ transporter MRP5 reduces $InsP_6$ levels in seeds and in other tissues (Nagy *et al*, 2009; Colombo *et al*, 2020). Notably, Arabidopsis *mrp5* mutants contain elevated levels of both $InsP_7$ and $InsP_8$ (Desai *et al*, 2014; Riemer *et al*, 2021). It has been speculated that higher cytosolic levels of $InsP_6$ in *mrp5* mutants may favor 5-$InsP_7$ (and thereby 1,5-$InsP_8$) synthesis by ITPKs and PPIK5Ks, but this has not been experimentally tested (Riemer *et al*, 2021). One could envision scenarios in which changing PP-InsP levels likewise impact $InsP_6$ pools, but this is difficult to test. Very recently, MRP5 was identified in a suppressor screen based on the *atitpk4* mutant, which has elevated levels of $InsP_3$ and reduced levels of $InsP_6$, $InsP_7$ and $InsP_8$ (Ren *et al*, 2024; Whitfield *et al*, 2023). This finding suggests that $InsP_6$ transport, or homeostasis and inositol polyphosphate metabolism are somehow linked, but the mechanism remains elusive (Ren *et al*, 2024). To this end, we are currently characterizing mutants identified in a genetic screen for PP-InsP function that seem to suggest a link between PP-InsP metabolism and inositol transport. For lack of robust experimental evidence we however feel that it is beyond the scope of this study to speculate about such

interactions. We have added the following paragraph to the discussion section (lines 428-433): "It is noteworthy, that not only PP-InsP levels, but also $InsP_6$ pools are affected in some of our genotypes (Figures 1F,3G; Supplementary Figures 5,8). The overaccumulation of $InsP_6$ in *nudt17/18/21* plants (Figure 1F), or the reduced $InsP_6$ levels in Mp*vip1*^{ge} (Figure 3G), cannot be explained by altered PP-InsP catabolism or biosynthesis in these mutants alone, given the much higher levels of $InsP_6$ compared to 1-$InsP_7$, 5-$InsP_7$ or 1,5-$InsP_8$. How PP-InsP may affect $InsP_6$ biosynthesis, transport or vacuolar storage remains to be investigated."

###

Minor points: can the authors make direct comparison between the kinetic parameters of the 'catabolic' activities measured here with the 'synthetic' activities of the enzymes reported elsewhere (and shown in Supplementary Figure 1.

author response

Thank you for this suggestion. To the best of our knowledge, no kinetic parameters have been reported for the putative ITPK and PPIP5K enzymes in Marchantia. Expression of the MpVIP1 kinase and phosphatase domains in baculovirus-infected insect cells resulted in severely aggregated protein samples in our hands, which could not be used in enzyme assays. The $InsP_6$ kinase activity of AtITKP1 has been estimated to be ~20 nmol min^{-1} mg^{-1} (Riemer *et al*, 2021). The 5-$InsP_7$ kinase activity of AtVIH2 is similar to the human HsPPIP5K2 enzyme, which is ~ 400 nmol min^{-1} mg^{-1} (Wang *et al*, 2012; Zhu *et al*, 2019). We have added the following statement to the discussion session (lines 377-383): "The specific activities are ~1400 nmol min^{-1} mg^{-1} and ~2600 nmol min^{-1} mg^{-1} for AtPFA-DSP1 and MpPFA-DSP1, respectively (Figures 1B and 3B). The specific activities for the phosphorylation of 5-$InsP_7$ to 1,5-$InsP_8$ by AtVIH2 and HsPPIKP2 were estimated to be ~ 400 nmol min^{-1} mg^{-1} (Wang *et al*, 2012; Zhu *et al*, 2019). ITPK1 generates 5-$InsP_7$ from $InsP_6$ with a specific activity of ~20 nmol min^{-1} mg^{-1} (Riemer *et al*, 2021). This suggests that in tissues expressing AtPFA-DSP1, or AtPFA-DSP2/4 (Figure 2A), 5-$InsP_7$ catabolism may impact 1,5-$InsP_8$ biosynthesis."

Otherwise, this is a very thorough study - executed with great skill.

author response

Thank you.

###

Reviewer #2: • This manuscript describes enzymes that are involved in the metabolism of inositol pyrophosphates.
• The demonstrate that PFA-SSP and NUDT proteins catalyze react ions in IP-PP metabolism.

• Plants that over express PFA-SSP and NUDT develop defective phenotypes. However, In Arabidopsis no defects were observed in loss of function mutants an higher order mutants were impossible to make. The authors concluded that gene redundancy may make it difficult to observe defective phenotypes in single mutants. Consequently, they generated lines carrying loss of function mutations in the homologous genes in Marchantia.

• PFA-SSP and NUDT mutants in Marchantia develop defective phenotypes. The morphology of the thallus is defective. The phenotype resembles phenotypes of plants with defective auxin signaling, however, not clear relationship to auxin could be found. This is not a problem, because many signaling pathways probably mutate to similar phenotypes.

• Given the role of IP-PPs in phosphate nutrition, the authors also examined phosphate nutrient responses in the mutants and found them to be defective. This suggests that the role of PFA-SSP and NUDT and by extension IP-PP is likely to be conserved among land plants.

• This is a detailed paper with valuable data.

One minor comment: the paper is difficult to read. While the language quality is fine, it is turgid reading. They authors could increase the impact of their paper but making the writing more accessible.

author response

We have revised the abstract, results and discussion section to make our finding more accessible.

###

Reviewer #3: PGENETICS-D-24-00432 Laurent et al.

SUMMARY: This manuscript defines the relative contributions of three different inositol pyrophosphate phosphatase families to plant PP-InsP catabolism and nutrient signaling using Arabidopsis and Marchantia as model systems. The approaches employed include biochemical characterization, overexpression and LOF phenotypic analyses, and assessments of altered function on PP-InsP levels Focus is trained on the Marchantia system where changes in cellular PP-InsP levels consistently result in phenotypes that include roles in phosphate signaling, nitrate homeostasis and cell wall biogenesis. Simultaneous removal of two phosphatase activities enhances the observed growth phenotypes. The authors conclude PPIP5K, PFA-DSP and NUDT inositol pyrophosphate phosphatases control these biological outcomes via modulation of plant PP-InsP pools.

GENERAL COMMENTS: This manuscript combines affinity purification approaches and biochemical and biological readouts to assess the roles of three different inositol pyrophosphate phosphatase families to plant PP-InsP homeostasis and nutrient signaling using Arabidopsis and Marchantia as model systems. The study solidly trods a rather standard formula of overexpression and LOF analyses to guage effects on Ins-PP pools, and associate those perturbations to biological function by phenotypic analyses and RNA-seq transcriptomics. The strengths of the MS are the general consistencies of the biochemical and Ins-PP pool data, and the fact that phenotypes are observed. The weaknesses are the authors do not go beyond rather phenomenological analyses to figure out the basis of any of the new phenotypes they describe. Given the wealth of data that already exist re Ins-PP biochemistry/biology in plants, it is the opinion of this reviewer that an opportunity to make a strong new contribution is missed.

SPECIFIC COMMENTS:

Reviewer #3 major point #1:

(i) Regarding phosphatase overexpression, and the Arabidopsis data in particular… What is the evidence that OE phenotypes are solely associated with the documented changes in Ins-PP pools? No other sugar pyrophosphate levels are effected by overexpression?

author response

Changes in PP-InsP levels result in altered phosphate starvation responses and phosphate starvation-induced gene expression (PSI) in Arabidopsis (Zhu *et al*, 2019; Dong *et al*, 2019; Riemer *et al*, 2021; Freed *et al*, 2022), which in turn leads to changes in nucleotide diphosphate, nucleotide triphosphate and carbohydrate levels, in order to redirect metabolism to reactions that do not require Pi or adenylates (Karthikeyan *et al*, 2007; Hammond & White, 2011; Zhu *et al*, 2019). Among the PSI genes there are acid phosphatases that remobilize P from phosphorylated or pyrophosphorylated cellular metabolites (Bustos *et al*, 2010; Zhu *et al*, 2019). Another PSI gene is UDP glucose phosphorylase (Ciereszko et al, 2005). These genes are differentially expressed comparing our different PFA-DSP and NUDT OX lines with the Col-0 wild-type control. Therefore, we did not attempt to quantify the levels of nucleotide phosphate or sugar phosphates, as they are likely to change in response to changes in the PP-InsP pool independent of the their putative role as alternative substrates as suggested by the reviewer. However, we have now quantified the levels of other inositol phosphates and polyphosphates (compare **reviewer #1 major points #2 and #3**) in revised Figures 1F, 3G and Supplementary Figs. 5A, 8A. In response to reviewer #1 (**reviewer #1 major point #1**) we provide additional experimental evidence that our PFA-DSP and NUDT enzymes are specific inositol pyrophosphate phosphatases.

However, we agree with reviewer #3 that it is difficult to mechanistically link *in vitro* enzyme activities to changes in cellular metabolite levels and associated phenotypes in planta. We have revised the following statements in the text accordingly (lines 56-57, changes in red) "...Loss-of-function mutants of the different enzymes show altered nitrate levels and changes in cell wall architecture, suggesting that inositol pyrophosphates may regulate cellular processes beyond phosphate homeostasis"

(lines 273-275) "Combined, Marchantia VIP1, PFA-DSP1 and NUDT1 are *bona fide* PP-InsP metabolizing enzymes *in vitro* and their genetic deletion alters PP-InsP pools *in planta*."

(lines 405-408) "Although no loss-of-function phenotypes for NUDT enzymes were observed, their induction under Pi starvation conditions suggests that these PP-InsP phosphatases may contribute to Pi homeostasis in Arabidopsis (Figure 2A, B).."

###

Reviewer #3 major point #2:

(ii) The authors rely on RNA-seq transcriptomics to gain insight but no attempts are made to validate those data independently by assessing levels or activities of key proteins of interest.

author response

There are no commercially available antibodies for the differentially expressed genes in our Col-0 +Pi vs -Pi, AtPFA-DSP2 OX, AtNUDT17 OX, nudt17/18/21 or Mp*pfa-dsp1*^{ge}, Mp*nudt1*^{ge}, Mp*vip1*^{ge} RNAseq experiments. We have previously validated the changes in protein accumulation for AtVIH1, AtVIH2 and SPX3 in response to changes in external Pi (Zhu *et al*, 2019) (compare Figure 2A,B,E).

Given the very large amount of time and resources it would take to validate our RNAseq experiments with custom made antibodies and recombinant enzyme preparations, we decided to follow the editor's recommendation to not explore this further (see editorial comments above).

###

Reviewer #3 major point #3:

(iii) The authors rely on RNA-seq data to implicate cell wall biogenesis as target of Ins-PP signaling but make little effort (other than rather crude histological stains) to investigate cell wall perturbations directly. This is a weakness.

author response

There are few reports that have investigated the composition and structure of wild-type *Marchantia polymorpha* cell wall using proteomics (Kolkas *et al*, 2022), histological stains (Kolkas *et al*, 2023), *in vitro* polysaccharide assays (Kolkas *et al*, 2023), immunogold labeling (Henry *et al*, 2020) and atomic force microscopy (Bonfanti *et al*, 2023). The histology method used in routine in our department's plant imaging unit is the sectioning of paraffin-embedded samples. However, this yielded insufficient preservation of Marchantia tissues (in particular, cell shrinking and poor preservation of the dorsal and ventral epidermis). As a result, for this study we developed and optimized Technovit preparations in the facility, which gave us far superior results regarding the quality of the preparation (Reviewer Figure 2).

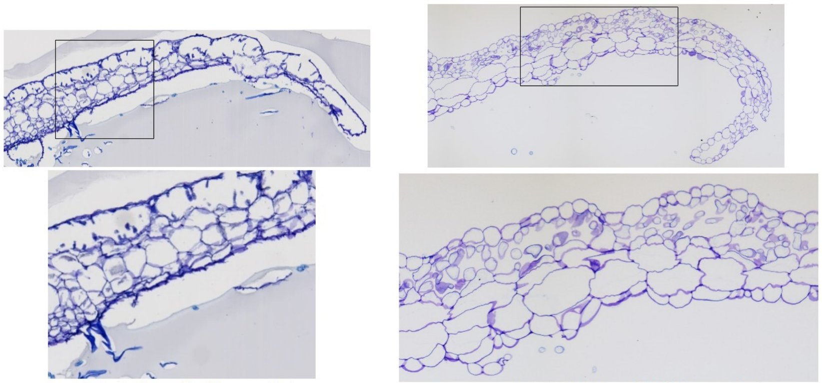

Embedded in **paraffin** (**12 µm section**) | Embedded in **Technovit 7100** (**5 µm section**)

Reviewer Figure 2

cross-sections of *Marchantia polymorpha* plants embedded in wax or resin, both stained by toluidine blue

One consequence of this change of embedding medium was that we had to optimize the different stainings conditions for Technovit-embedded samples. This took considerable amounts of time. We did not engage with more advanced techniques such as immunogold labeling because of the long time this technique takes to set up, and because of the lack of suitable cell wall mutants in Marchantia, which would be needed to control such experiments. We have however extended our histological analysis to additionally include nile red staining for suberin and putative lipid bodies (Piccinini *et al*, 2024) (revised Figure 5C and D) The revised statement in the text reads (lines 318-322 , changes in red):"Fluorol yellow staining for lipidic compounds such as suberin or cutin also showed strong signals in the dorsal and ventral epidermal layers, while nile red staining showed a reduced signal in the parenchymatous cells (Figure 5E), suggesting that the Mp*pfa-dsp1*^{ge} and Mp*vip1*^{ge} mutants may contain higher levels of polyester cell wall polymers in the epidermis."

The method section and the Figure 5 figure legend have been updated accordingly.

###

Reviewer #3 major point #4:

(iv) The authors themselves state: '…alterations in nitrogen supply affect cell wall organization and composition in several plant species (Fernandes et al., 2013; Rivai et al., 2021; Głazowska et al., 2019), providing an alternative rationale for the cell wall defects observed in our Mppfa-dsp1ge and Mpvip1ge mutants (Figure 5)." Why not test this directly?

author response

Thank you for suggesting this experiment. For the revision of this manuscript, we grew plants derived from single gemma on plates containing B5 medium for one week, and an additional week in either B5 medium, or in B5 where KNO_3 was replaced by KCl. We found increased ruthenium red (overall) and fluorol yellow (epidermis) staining in Tak-1 wild-type plants grown in nitrate starvation when compared to the untreated control (Reviewer Figure 3 A,B). Renaissance SR2200 staining was reduced in nitrate-starved Tak-1 plants (overall) when compared to the nitrate sufficient control (Reviewer Figure 3C). This suggests that changes in cell wall composition occur in Marchantia plants experiencing nitrate limitation. However, also our Mp*pfa-dsp1*^{ge} and Mp*vip1*^{ge} mutants show increased ruthenium red staining (overall) very similar to the Tak-1 wild type (Reviewer Figure 3A). In the case of fluorol yellow, there seems to be a stronger staining of the epidermides in different Mp*pfa-dsp1*^{ge} plants, but overall less staining in Mp*vip1*^{ge} mutant plants (Reviewer Figure 3B). However, there is significant variation among different the samples tested. We conclude from these pilot experiments that nitrogen starvation induced changes in cell wall composition could be altered in our mutants. However, given that there are no consistent changes in the different mutants this issue will need to be investigated in much more detail in future studies.

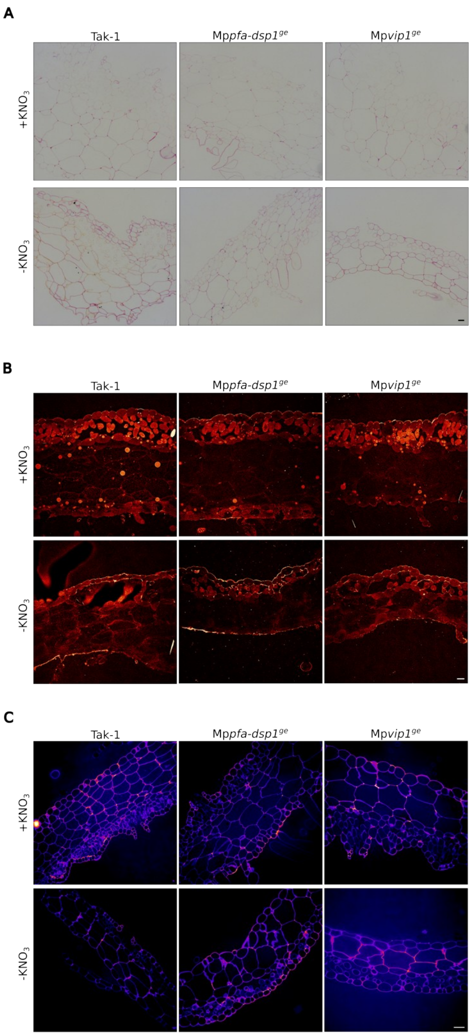

Reviewer Figure 3 Nitrate starvation is altering the cell wall composition of Marchantia.

(A-C) 2-week-old Tak-1, Mp*pfa-dsp1*^{ge} and Mp*vip1*^{ge} plants were grown from gemmae on either no nitrate (-KNO_3, media was supplemented with 24 mM KCl) or in 24 mM KNO_3 (+KNO_3). **(A)** Ruthenium red-stained cross-sections of Tak-1, Mp*pfa-dsp1*^{ge} and Mp*vip1*^{ge} plants (scale bar= 50 µm). **(B)** Enlarged view of fluorol yellow-stained cross-sections of Tak-1, Mp*pfa-dsp1*^{ge} and Mp*vip1*^{ge} plants (scale bar= 40 µm). **(C)** Enlarged view of Renaissance SR2200-stained cross-sections of Tak-1, Mp*pfa-dsp1*^{ge} and Mp*vip1*^{ge} plants (scale bar= 50 µm).

References

Bonfanti A, Smithers ET, Bourdon M, Guyon A, Carella P, Carter R, Wightman R, Schornack S, Jönsson H & Robinson S (2023) Stiffness transitions in new walls post-cell division differ between Marchantia polymorpha gemmae and Arabidopsis thaliana leaves. *Proc Natl Acad Sci U S A* 120: e2302985120

Bustos R, Castrillo G, Linhares F, Puga MI, Rubio V, Pérez-Pérez J, Solano R, Leyva A & Paz-Ares J (2010) A Central Regulatory System Largely Controls Transcriptional Activation and Repression Responses to Phosphate Starvation in Arabidopsis. *PLoS Genet* 6: e1001102

Ciereszko I, Johansson H & Kleczkowski LA (2005) Interactive effects of phosphate deficiency, sucrose and light/dark conditions on gene expression of UDP-glucose pyrophosphorylase in *Arabidopsis*. *Journal of Plant Physiology* 162: 343–353

Colombo F, Paolo D, Cominelli E, Sparvoli F, Nielsen E & Pilu R (2020) MRP Transporters and Low Phytic Acid Mutants in Major Crops: Main Pleiotropic Effects and Future Perspectives. *Front Plant Sci* 11: 1301

Desai M, Rangarajan P, Donahue JL, Williams SP, Land ES, Mandal MK, Phillippy BQ, Perera IY, Raboy V & Gillaspy GE (2014) Two inositol hexakisphosphate kinases drive inositol pyrophosphate synthesis in plants. *Plant J* 80: 642–653

Dong J, Ma G, Sui L, Wei M, Satheesh V, Zhang R, Ge S, Li J, Zhang T-E, Wittwer C, *et al* (2019) Inositol Pyrophosphate InsP8 Acts as an Intracellular Phosphate Signal in Arabidopsis. *Molecular Plant*

Freed C, Craige B, Cridland C, Donahue J, Williams SP, Kim J & Gillaspy G (2022) Translational approach to increase phosphate accumulation in two plant species through perturbance of inositol pyrophosphates Plant Biology

Garza JA, Ilangovan U, Hinck AP & Barnes LD (2009) Kinetic, Dynamic, Ligand Binding Properties, and Structural Models of a Dual-Substrate Specific Nudix Hydrolase from Schizosaccharomyces pombe. *Biochemistry* 48: 6224–6239

Gaugler P, Schneider R, Liu G, Qiu D, Weber J, Schmid J, Jork N, Häner M, Ritter K, Fernández-Rebollo N, *et al* (2022) Arabidopsis PFA-DSP-Type Phosphohydrolases Target Specific Inositol Pyrophosphate Messengers. *Biochemistry* 61: 1213–1227

Hammond JP & White PJ (2011) Sugar Signaling in Root Responses to Low Phosphorus Availability1. *Plant Physiol* 156: 1033–1040

Henry JS, Lopez RA & Renzaglia KS (2020) Differential localization of cell wall polymers across generations in the placenta of Marchantia polymorpha. *J Plant Res* 133: 911–924

Ingram SW, Stratemann SA & Barnes LD (1999) Schizosaccharomyces pombe Aps1, a Diadenosine 5‘,5‘ ‘‘-P1,P6-Hexaphosphate Hydrolase That Is a Member of the Nudix (MutT) Family of Hydrolases: Cloning of the Gene and Characterization of the Purified Enzyme,. *Biochemistry* 38: 3649–3655

Karthikeyan AS, Varadarajan DK, Jain A, Held MA, Carpita NC & Raghothama KG (2007) Phosphate starvation responses are mediated by sugar signaling in Arabidopsis. *Planta* 225: 907–918

Kilari RS, Weaver JD, Shears SB & Safrany ST (2013) Understanding inositol pyrophosphate metabolism and function: kinetic characterization of the DIPPs. *FEBS Lett* 587

Kolkas H, Balliau T, Chourré J, Zivy M, Canut H & Jamet E (2022) The Cell Wall Proteome of Marchantia polymorpha Reveals Specificities Compared to Those of Flowering Plants. *Frontiers in Plant Science* 12

Kolkas H, Burlat V & Jamet E (2023) Immunochemical Identification of the Main Cell Wall Polysaccharides of the Early Land Plant Marchantia polymorpha. *Cells* 12: 1833

Laha NP, Giehl RFH, Riemer E, Qiu D, Pullagurla NJ, Schneider R, Dhir YW, Yadav R, Mihiret YE, Gaugler P, *et al* (2022) INOSITOL (1,3,4) TRIPHOSPHATE 5/6 KINASE1-dependent inositol polyphosphates regulate auxin responses in Arabidopsis. *Plant Physiol* 190: 2722–2738

Lonetti A, Szijgyarto Z, Bosch D, Loss O, Azevedo C & Saiardi A (2011) Identification of an evolutionarily conserved family of inorganic polyphosphate endopolyphosphatases. *J Biol Chem* 286: 31966–31974

Márquez-Moñino MÁ, Ortega-García R, Shipton ML, Franco-Echevarría E, Riley AM, Sanz-Aparicio J, Potter BVL & González B (2021) Multiple substrate recognition by yeast diadenosine and diphosphoinositol polyphosphate phosphohydrolase through phosphate clamping. *Sci Adv* 7: eabf6744

Nagy R, Grob H, Weder B, Green P, Klein M, Frelet-Barrand A, Schjoerring JK, Brearley C & Martinoia E (2009) The Arabidopsis ATP-binding cassette protein AtMRP5/AtABCC5 is a high affinity inositol hexakisphosphate transporter involved in guard cell signaling and phytate storage. *J Biol Chem* 284: 33614–33622

Piccinini L, Nirina Ramamonjy F & Ursache R (2024) Imaging plant cell walls using fluorescent stains: The beauty is in the details. *Journal of Microscopy* 295: 102–120

Ren Y, Jiang M, Zhu J-K, Zhou W & Zhao C (2024) Simultaneous mutations in ITPK4 and MRP5 genes result in a low phytic acid level without compromising salt tolerance in Arabidopsis. *J Integr Plant Biol*

Riemer E, Qiu D, Laha D, Harmel RK, Gaugler P, Gaugler V, Frei M, Hajirezaei M-R, Laha NP, Krusenbaum L, *et al* (2021) ITPK1 is an InsP6/ADP phosphotransferase that controls phosphate signaling in Arabidopsis. *Molecular Plant* 14: 1864–1880

Safrany ST, Ingram SW, Cartwright JL, Falck JR, McLennan AG, Barnes LD & Shears SB (1999) The Diadenosine Hexaphosphate Hydrolases fromSchizosaccharomyces pombe and Saccharomyces cerevisiae Are Homologues of the Human Diphosphoinositol Polyphosphate Phosphohydrolase. *Journal of Biological Chemistry* 274: 21735–21740

Sanchez AM, Schwer B, Jork N, Jessen HJ & Shuman S (2023) Activities, substrate specificity, and genetic interactions of fission yeast Siw14, a cysteinyl-phosphatase-type inositol pyrophosphatase. *mBio* 14: e02056-23

Steidle EA, Chong LS, Wu M, Crooke E, Fiedler D, Resnick AC & Rolfes RJ (2016) A Novel Inositol Pyrophosphate Phosphatase in Saccharomyces cerevisiae. *Journal of Biological Chemistry* 291: 6772–6783

Steidle EA, Morrissette VA, Fujimaki K, Chong L, Resnick AC, Capaldi AP & Rolfes RJ (2020) The InsP7 phosphatase Siw14 regulates inositol pyrophosphate levels to control localization of the general stress response transcription factor Msn2. *J Biol Chem* 295: 2043–2056

Stevenson-Paulik J, Bastidas RJ, Chiou S-T, Frye RA & York JD (2005) Generation of phytate-free seeds in *Arabidopsis* through disruption of inositol polyphosphate kinases. *Proc Natl Acad Sci USA* 102: 12612–12617

Wang H, Falck JR, Hall TMT & Shears SB (2012) Structural basis for an inositol pyrophosphate kinase surmounting phosphate crowding. *Nat Chem Biol* 8: 111–116

Wang H, Gu C, Rolfes RJ, Jessen HJ & Shears SB (2018) Structural and biochemical characterization of Siw14: A protein-tyrosine phosphatase fold that metabolizes inositol pyrophosphates. *Journal of Biological Chemistry* 293: 6905–6914

Wang H, Perera L, Jork N, Zong G, Riley AM, Potter BVL, Jessen HJ & Shears SB (2022) A structural exposé of noncanonical molecular reactivity within the protein tyrosine phosphatase WPD loop. *Nat Commun* 13: 2231

Whitfield HL, He S, Gu Y, Sprigg C, Kuo H-F, Chiou T-J, Riley AM, Potter BVL, Hemmings AM & Brearley CA (2023) Diversification in the inositol tris/tetrakisphosphate kinase (ITPK) family: crystal structure and enzymology of the outlier AtITPK4. *Biochemical Journal* 480: 433–453

Zhu J, Lau K, Puschmann R, Harmel RK, Zhang Y, Pries V, Gaugler P, Broger L, Dutta AK, Jessen HJ, *et al* (2019) Two bifunctional inositol pyrophosphate kinases/phosphatases control plant phosphate homeostasis. *eLife* 8: e43582

Zong G, Jork N, Hostachy S, Fiedler D, Jessen HJ, Shears SB & Wang H (2021) New structural insights reveal an expanded reaction cycle for inositol pyrophosphate hydrolysis by human DIPP1. *The FASEB Journal* 35: e21275
